# Ozonation of carbamazepine and its main transformation products: product determination and reaction mechanisms

**DOI:** 10.1007/s11356-020-08795-0

**Published:** 2020-04-25

**Authors:** Matilda Kråkström, Soudabeh Saeid, Pasi Tolvanen, Narendra Kumar, Tapio Salmi, Leif Kronberg, Patrik Eklund

**Affiliations:** 1 Laboratory of Organic Chemistry, Johan Gadolin Process Chemistry Centre, Åbo Akademi University, Biskopsgatan 8, FI-20500 Åbo/Turku, Finland; 2 Laboratory of Industrial Chemistry and Reaction Engineering, Johan Gadolin Process Chemistry Centre, Åbo Akademi University, Biskopsgatan 8, FI-20500 Åbo/Turku, Finland

**Keywords:** Wastewater treatment, Ozonation, Pharmaceuticals, Carbamazepine, Transformation products, Product identification, Quantification

## Abstract

Carbamazepine (CBZ) is a recalcitrant pharmaceutical often detected in wastewater and in the environment. CBZ can be removed from wastewater through advanced oxidation treatment methods such as ozonation. In this study, CBZ and its transformation product 1-(2-benzaldehyde)-(1H,3H)-quinazoline-2,4-dione (BQD) were ozonated, and the formation and transformation of their ozonation products were investigated using liquid chromatography coupled to ion trap mass spectrometry and high-resolution mass spectrometry as well as nuclear magnetic resonance (NMR). The main products, 1-(2-benzaldehyde)-4-hydro-(1H,3H)-quinazoline-2-one (BQM) and BQD were quantified using isolated standards and LC-UV. Of the original CBZ concentration, 74% was transformed into BQM and 83% of BQM was further transformed into BQD. Both products are more stable than CBZ and could still be detected after 240 min of ozonation. Another major product, 2,2′-azanediyldibenzaldehyde (TP225) was for the first time identified using NMR. Twelve further CBZ products were identified.

**Electronic supplementary material:**

The online version of this article (10.1007/s11356-020-08795-0) contains supplementary material, which is available to authorized users.

## Introduction

Carbamazepine (CBZ), an antiepileptic pharmaceutical, has a tricyclic dibenzazepine structure consisting of two benzene rings fused to an azepine group. In a study where the occurrence of pharmaceuticals in the Baltic Sea was studied, CBZ was the most frequently detected compound with a detection frequency of 86% (Björnelius et al. 2018). The concentration of CBZ in the Baltic Sea is in the ng/L range (Björnelius et al. 2018).

Carbamazepine, along with other pharmaceuticals, enters the aquatic environment via wastewater treatment plants (WWTPs). Carbamazepine does not adsorb to sludge (Hörsing et al. 2011). In conventional-activated sludge processes, the removal efficiency of CBZ is between − 67 and 11% (Verlicchi et al. 2012). In wastewater treatment plants, the transformation rate of CBZ is usually below 20% (Zhang et al. 2008). The concentration of CBZ in effluent water from WWTPs is often higher than in the influent leading to a negative removal rate (Björnelius et al. 2018; UNESCO and HELCOM 2017; Vieno et al. 2006). This is believed to be due to the deconjugation of CBZ that takes place in WWTPs (Verlicchi et al. 2012). CBZ is very stable in the environment. During direct photolysis experiments, the half-life of CBZ is between 84 h and 87.5 days (Yamamoto et al. 2009; Andreozzi et al. 2002). The biotransformation half-life is between 125 and 315 days (Yamamoto et al. 2009; Durán-Álvarez et al. 2015). In field tests, the half-life has been found to be much longer: the half-lives of CBZ in a Swedish lake and in the Baltic Sea are both around 3.5 years (Björnelius et al. 2018; Zou et al. 2015).

Due to the high persistence of CBZ in the environment and its recalcitrance in the activated sludge wastewater treatment process, new methods have been proposed for the removal of CBZ from wastewater. Several advanced oxidation treatment methods have been investigated for the removal of CBZ, including ozonation, ultraviolet light and hydrogen peroxide, Fenton and photo-Fenton processes, photocatalysis, and ultrasonic irradiation (Mohaparta et al. 2014). Ozonation is one of the most efficient transformation methods, usually with a removal efficiency of 100% (Mohaparta et al. 2014).

Ozonation involves the transformation of organic compounds with the aid of direct reactions with ozone molecules or indirectly via the formation of hydroxyl radials which can in turn react with organic compounds such as CBZ. Ozone reacts selectively with carbon-carbon double bonds, aromatic rings, and functional groups containing nitrogen or oxygen (Ikehata et al. 2006). Hydroxyl radicals react non-selectively through hydrogen abstraction, radical-radical reactions, electrophilic addition, and electron transfer (Ikehata et al. 2006).

The removal of CBZ does not necessarily result in the removal of toxicity. Although Andreozzi (2002) found that neither ozonated nor non-ozonated CBZ had any inhibiting effect on the micro-algae *Raphidocelis subcapitata* or *Botryococcus braunii*; others have found that the transformation of CBZ leads to increased toxicity. The human metabolite of CBZ acridine-9-carbaldehyde has been shown to be more cytotoxic to lymphocytes than CBZ (Furst and Uetrecht 1995). The CBZ phototransformation products acridine and acridone are more toxic than CBZ to the bacteria *Vibrio fischeri*, the algae *Pseudokirchneriella subcapitata*, and the cladoceran *Daphnia magna* (Donner et al. 2013). During ozonation of CBZ, the toxicity to the gram-negative bacteria *Aliivibrio fischeri* was proportional to the concentration of two of the transformation products (Azaïs et al. 2017). This indicates that not only the removal of CBZ has to be investigated but also the formation of products is very important, since the elimination of the transformation products is at least as important as the elimination of CBZ.

In humans, CBZ is metabolized via epoxidation, hydroxylation, the formation of an iminoquinone product, rearrangements to 9-acridine carboxyaldehyde, acridine and acridone, and addition of a glucuronide (Furst and Uetrecht 1995; Breton et al. 2005; Martins et al. 2018). The main metabolites are dihydroxy-carbamazepine and carbamazepine epoxide (Jiang et al. 2019). In nature, CBZ undergoes phototransformation leading to the formation of an epoxide, hydroxlation products, and rearrangements to acridine and acridone (Chiron et al. 2006; de Laurentiis et al. 2012). Phototransformation can also result in the formation of the rearrangement product 1-(2-benzaldehyde)-(1H,3H)-quinazoline-2,4-dione (BQD) (de Laurentiis et al. 2012). CBZ can undergo biotransfomation in soil, leading to the formation of an epoxide, hydroxylation products, and rearrangement to acridine (Li et al. 2013). Advanced oxidation treatment mainly leads to the formation of 1-(2-benzaldehyde)-4-hydro-(1H,3H)-quinazoline-2-one (BQM), which is further transformed to BQD (McDowell et al. 2005). While the main reaction pathway of CBZ and ozone has been investigated, uncertainties are left in the ozonation reaction of CBZ, and therefore, it is important to study the fate of the products more in detail. For example, a product with [M + H]^+^ = 226 or M^+^ = 225 and fragments with m/z = 208, 198 and 180 has been detected in several different studies. However, no conclusive structure of this product has been presented (Kosjek et al. 2009; Gebhardt and Schröder 2007; Hu et al. 2009; Li et al. 2011; Pan et al. 2017).

When developing a novel wastewater treatment method, one needs to consider not only the removal of the target pollutant but also the transformation products formed during the treatment. This can assist in choosing the optimal treatment method for the removal of the pharmaceutical. One aim of this study was to conclusively determine the structures of all of the major products formed when CBZ is ozonated. The second aim of our study was to quantify the major transformation products and study their fate in the ozonation. The third aim of this study was to ozonate BQD, the major transformation formed during the ozonation of CBZ, in order to determine its stability in comparison with CBZ and to identify the products which are formed from BQD.

## Materials and methods

### Materials

Carbamapezine was purchased from Sigma Life Science. Methanol was obtained from VWR (France). The water used in the LC-MS analysis was purified using a Millipore Simplicity 185 system (Millipore S.A.S., Molsheim, France). The acetonitrile used in the LC-MS analysis was of LC-MS grade and was obtained from Fisher Scientific, while formic acid was obtained from Sigma-Aldrich.

### Ozonation experiments

The method used for the ozonation experiments is presented in Saeid et al. (2020). Briefly, the ozonation experiments were conducted in a double jacket isotermal glass reactor. The reactor was operated in semibatch mode and the temperature was 20 °C. Ozone was provided with an ozone generator (Absolute Ozone, Nano model, Canada). The inlet ozone gas flow was 21 mg/L determined by iodine volumetric titration method, and the dissolved ozone concentration in the reactor was 0.44 mg/L determined by the indigo method as described in Saeid et al. 2018. The feed gas consisted of oxygen (109.5 mL/min) and nitrogen (1.5 mL/min). Nitrogen was used in the feed gas for better performance of the ozonator in accordance with the ozonator manual. For the CBZ experiments, the stock solution was prepared by dissolving CBZ in methanol to give a concentration of 3 g/L. For the ozonation experiments, stock solution (10 mL) was added to de-ionized water (1 L). The initial concentration of CBZ was 30 mg/L and the reaction time was 240 min. BQD was found to react with methanol, so the stock solution for the BQD experiments was prepared in acetonitrile. The initial concentration of BQD was 6 mg/L, and the reaction time was 240 min.

### Structure determination and quantification

The structures of transformation products and the concentrations of CBZ and its transformation products were determined according to Saeid et al. (2020). For structure determination, an Agilent 1100 LC/MSD ion trap mass spectrometer equipped with an electrospray ionization (ESI) source was used in full scan and MS^2^ scan modes. Both positive and negative ionization modes were used. Nitrogen and argon were used as drying gas and collision gas, respectively. The scan range was 50–600 m/z. The acquisition parameters can be found in Table S1 (supplementary material). For the chromatographic separation, an Agilent 1100 system was used. The same LC method was used for both ionization modes. The system consisted of a binary pump, a vacuum degasser, an autosampler, a thermostatted column oven, and a variable wavelength detector. The temperature of the column (Waters Atlantis T3 C18, 2.1 × 100 mm, 3 μm) was set to 30 °C and was used, and the variable wavelength detector was set to 254 nm. For the elution, a gradient program was used. Eluent A consisted of 0.1% formic acid in water, and eluent B consisted of 0.1% formic acid in acetonitrile. The initial eluent composition was 0% (B) for 1 min, followed by a linear increase to 30% (B) over 9 min. The composition was further increased linearly to 95% (B) over 14 min. Finally, the column was equilibrated by returning to the initial conditions over the next 1 min and remaining at 0% (B) for 10 min. The flow rate was 0.3 mL/min, and the injection volume was 30 μL. A commercial standard was used for the quantification of CBZ. BQM and BQD were quantified using synthesized standards. Six-point calibration curves with concentrations between 0.5 and 50 mg/L were prepared in water by diluting the standards. LC-UV was used to quantify CBZ and its products. The method validation is presented in supplementary information. The same LC method was used for the structure determination and quantification.

High-resolution mass spectra of the products were obtained using a Bruker Daltonics micrOTOF quadrupole and time-of-flight mass spectrometer equipped with an electrospray ionization (ESI) source. The instrument was operated in full scan mode. Positive ionization mode was used. The scan range was 50–1100 m/z. The acquisition parameters can be found in Table S1 (supplementary material). Argon was used as drying gas and collision gas. The chromatographic separation was performed using an Agilent 1200 binary pump equipped with a vacuum degasser, an autosampler, a thermostatted column oven, and a diode array detector. The column and chromatographic methods were the same as above.

### Synthesis of BQM and BQD

In order to ascertain the structures of the main products, the method presented in the “Ozonation experiments” section was used to prepare a sample, except that the concentration was 100 mg/L and the ozonation was stopped after 30 min. Subsequently, the sample was evaporated in a rotavapor. After evaporation, the sample was dissolved in chloroform, and 200 mg Celite was added. The sample was evaporated and transferred to a RediSep Rf Teledyne ISCO cartridge (5 g). The chromatographic separation was performed using automated flash system (CombiFlash EZ prep, Teledyne ISCO) equipped with a Redi Sep Rf Gold HP silica column (4 g) and using ethyl acetate:petrol ether as the eluent system (gradient program). After separation, the fractions were evaporated and analyzed using ^1^H NMR and ^13^C NMR experiments run on a MHz Bruker AVANCE-III NMR-system with a liquid nitrogen-cooled Prodigy BBO probe.

### Product identification workflow

Initially, the samples were analyzed using an ion trap mass spectrometer operating in auto MS^5^ mode. The mass spectra for all the major peaks in the UV chromatogram were investigated. The results were compared with previously published results. Published literature contained conflicting information concerning the fragments of some of the peaks, so they were tentatively identified based on the most likely fragmentation pattern. For previously unidentified products, the likely fragmentation patterns of potential products were determined and compared with the experimentally obtained results. Since some uncertainty remained concerning the identification of the products, NMR experiments were performed on products isolated with flash chromatography. NMR together with high-resolution mass spectrometry could be used to conclusively identify three of the main products. Finally, high-resolution mass spectrometry was performed in order to provide more certainty to the identification of the products. The confidence level of the products was assigned based on Schymanski et al. (2014); however, some modifications were necessary since the Schymanski method was developed for HRMS data. The compounds are divided into four confidence levels. Level one represents compounds whose structures are confirmed with reference standards of NMR analysis. Level two represents probable structures based on matching literature or database searches (level 2a) or when no other structure fits the observed fragmentation pattern (level 2b). Level three represents tentative candidates for which the exact structure remains speculative. Level four represents compounds for which the structure is completely unknown.

## Results and discussion

### Identification of CBZ transformation products

### Previously identified CBZ oxidation products

The two main CBZ ozonation products are BQM and BQD. Both could be identified with confidence level 1 based on NMR spectra. Other previously detected CBZ ozonation products include the hydroxylated derivatives formed from BQM and BQD: BaQM and BaQD. These products are further discussed in the supplementary information. Also, TP239, TP223, TP146, and TP162 have been identified as CBZ ozonation products. These products were identified with confidence level 2a based on the comparison with existing literature. Due to the presence of methanol in the experiments, it was possible to detect methoxylated versions of BQM and BQD. These products are also formed during biotransformation (Golan-Rozen et al. 2015).

One of the major products, TP225, had the retention 26.9 min and [M + H]^+^ = 226 (Table 1). The MS^2^ scan showed major fragments with m/z = 208, m/z = 206, and m/z = 180. Products with similar mass spectra have been detected previously (Kosjek et al. 2009; Gebhardt and Schröder 2007; Hu et al. 2009; Li et al. 2011); however, several possible structures have been presented, and the structure of this product has not previously been confirmed using NMR.

**Table 1:**
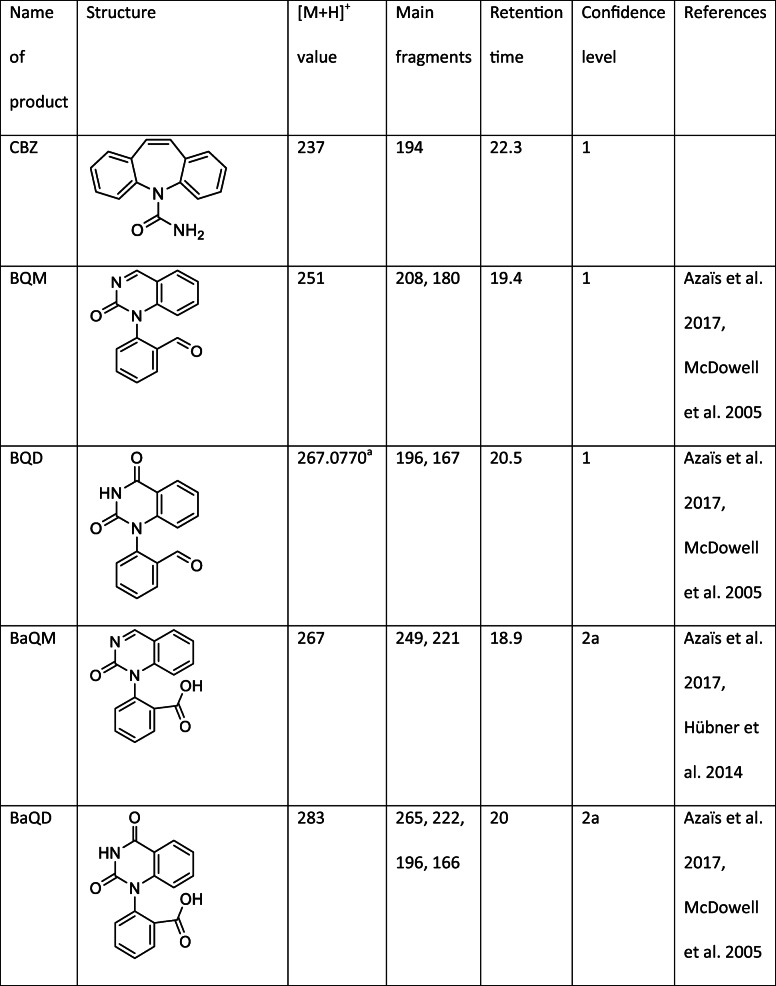

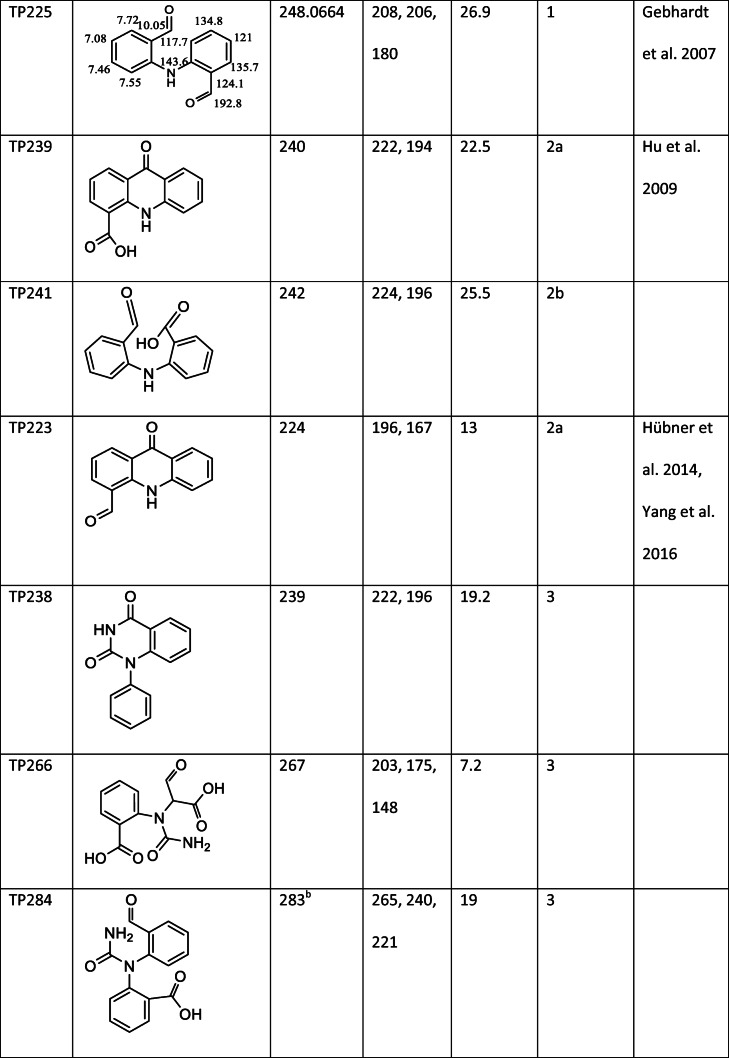

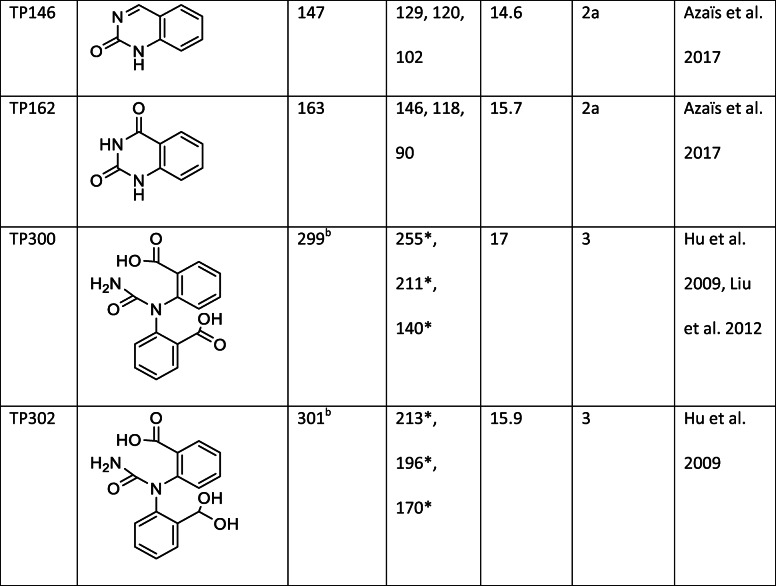
CBZ and identified CBZ transformation products

^a^Accurate mass

^b^Measured in negative mode

In order to further investigate the structure of TP225, the compound was isolated using the same method as for BQM and BQD. The high-resolution mass of the product was m/z = 248.0664, which corresponds to the empirical formula C_14_H_11_NNaO_2_ (error 9 ppm). The structure of the compound was confirmed by ^1^H NMR, COSY, ^13^C NMR, HMBC, and HSQC experiments. The NMR results are discussed in detail in the supplementary information. The NMR spectrum showed peaks from two different structures present with the ratio 1:1. One of the structures was symmetrical. Based on the mass spectrum and the NMR data, it was concluded that the product is 2,2′-azanediyldibenzaldehyde (TP225, Table 1). The proton and carbon shifts associated with this structure can be seen in Table 1. The identification of the second products, a methylated derivative of TP225, is presented in supplementary information. TP225 could be identified with confidence level 1.

### CBZ oxidation products not previously published

TP 241 had a retention time of 25.5 min and [M + H]^+^ = 242. The MS^2^ scan showed two fragments, one with m/z = 224, corresponding to the loss of an OH group and the other one with m/z = 196 corresponding to the loss of a COOH group. This could correspond to 2-(2-formylanilino)benzoic acid (Table 1). TP241 was identified with confidence level 2b.

TP238 had the retention time of 19.3 min and [M + H]^+^ = 239. The odd value for the [M + H]^+^ peak indicated that both nitrogen atoms are still attached to the structure. The fragmentation of TP238 was similar to that of BQD and BaQD. The main fragment of both BQD and TP238 had m/z = 196. This fragment was a result of the loss of NHCO in TP238 and NHCO and CHO in BQD. Additionally, the minor fragment in TP238 (m/z = 222) was the same as the major fragment of BaQD. This fragment corresponded to the loss of an NH_2_ group in TP238 and NH_2_ and COOH in BaQD. These similarities indicated that the structures of the products were similar, except that TP238 has neither an aldehyde nor a carboxylic acid group (Table 1). The structure is still speculative, so the product was identified with confidence level 3.

TP266 had [M + H]^+^ = 267, the same value as BQD and BaQM; however, the retention time (7.2 min) was very different from the retention times of BQD and BaQM (20.5 and 18.9 min, respectively), and the fragments were completely different. This product was formed only towards the end of the experiment. The main fragment m/z = 203 corresponded to the loss of COOH, and rearrangement and loss of OH. MS^3^ scans showed further fragmentation of the main fragment (Fig. S10). TP266 is probably formed when opening one of the aromatic rings. A tentative structure for this product was obtained at confidence level 3 and is displayed in Table 1.

TP284 had retention time 19 min and [M−H]^−^ = 283. The major fragments had m/z = 265, corresponded to the loss of an OH group, m/z = 240, corresponded to the loss of CONH_2_, and m/z = 221, corresponded to the loss of both OH and CONH_2_. This indicates that the heteroaromatic ring had been broken with the addition of an OH group. The OH group could be attached to either of the available carbons. A tentative structure for this product was obtained at confidence level 3 and is displayed in Table 1.

**Fig. 1:**
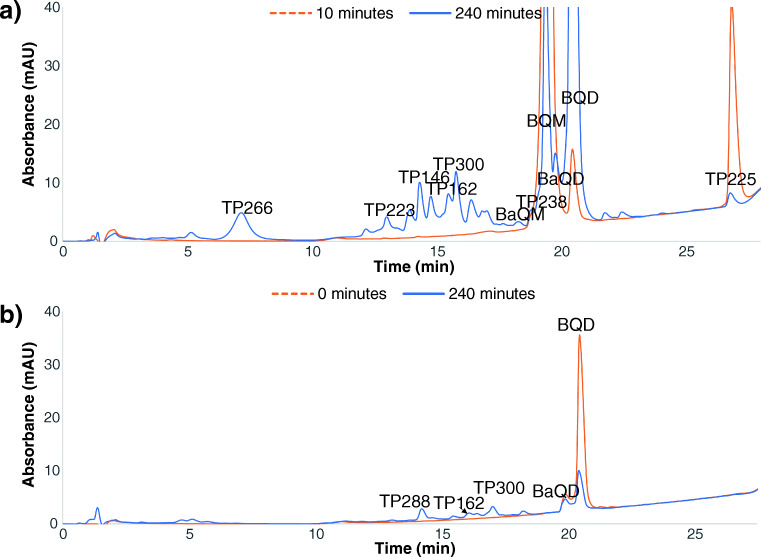
UV chromatograms (**a**) of a CBZ ozonation sample taken after 10 min (dashed line) and 240 min (solid line) of ozonation and (**b**) of a BQD sample taken after 0 min (dashed line) and 240 min (solid line) of ozonation

Ozonation of CBZ initially led to the formation of BQM and TP225, followed by BQD. After 10 min, only these three major products could be detected (Fig. 1a). After 240 min, also other minor products could be detected (Fig. 1a).

### Identification of BQD transformation products

The transformation of the CBZ transformation product BQD was investigated by performing experiments in which pure BQD was ozonated directly. During the 240 min experiment, 82% of BQD was transformed. BaQD could be detected during the whole experiment, including the unreacted sample. The UV peak area corresponding to BaQD remained unchanged during the experiment. The carboxylic acid group in BaQD is formed from the aldehyde in BQD. BaQD might be formed when BQD is dissolved in water under oxidative conditions.

Several transformation products with a shorter retention time than BQD could be detected (Fig. 1b). The three major products were TP288, TP162, and TP300 (Fig. 1b). TP162 was also detected as a third-generation transformation product during the ozonation of CBZ. TP288, TP300, and TP302 were not detected during the ozonation of CBZ, likely since the experiments ended before these products were formed in high-enough concentrations to be detected.

TP300 and the minor product TP302 are formed from CBZ during dielectric barrier discharge oxidation (Liu et al. 2012) and catalytic oxidation (Hu et al. 2009); however, no MS fragmentation data have been published. In this study, TP300 had a retention time 17 min and [M−H]^−^ = 299. The mass spectrum and fragmentations are presented in Fig. S14 (supplementary information). This product is formed from BaQD via a hydroxylation reaction. The OH group could be attached to either of the available carbons. TP302 had a retention time 15.9 min and [M−H]^−^ = 301. The MS^2^ scan showed fragments with m/z = 213, 196, and 170. The structure of TP302 is similar to that of TP300, but TP302 contains a geminal diol group instead of a carboxylic acid group. Both products were identified with confidence level 3 and possible structures are presented in Table 1.

TP288 could be detected in both negative and positive modes. The major fragments in positive mode were m/z = 228, 202, and 184. The major fragments in negative mode are m/z = 216, 172, and 156. The structure of this product is not known (confidence level 4).

### Transformation pathway

**Fig. 2:**
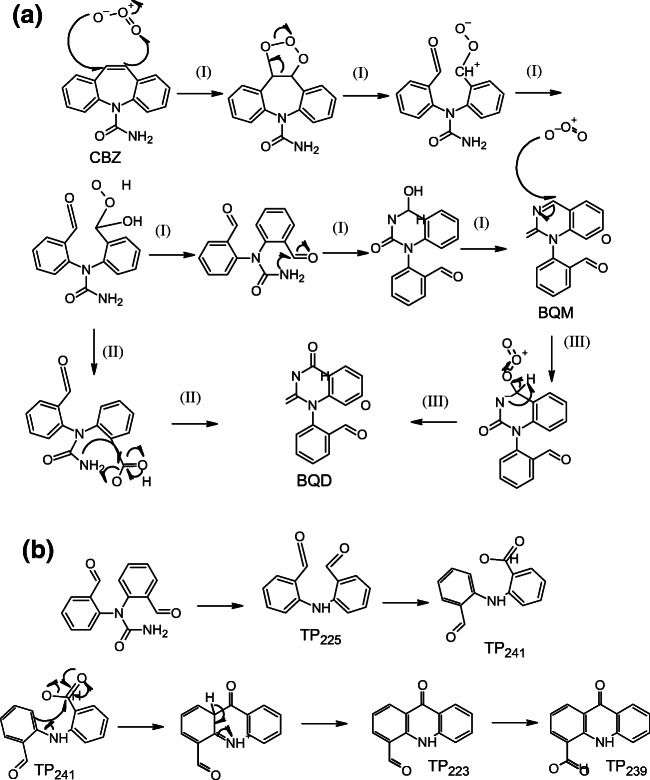
Transformation pathways leading to the formation of (**a**) BQM and BQD and (**b**) TP239

CBZ can react with ozone via a Criegee mechanism (McDowell et al. 2005, Hübner et al. 2014, Liu et al. 2012, Yang et al. 2016) forming two aldehyde groups. The amine attacks the aldehyde to form an imine leading to the formation of BQM, which could react further with ozone forming BQD (Fig. 2a pathway (I)). The mechanism for the ozonation of BQM has not been published. However, it is likely that ozone reacted with BQM via a nucleophilic attack by ozone to the imine type carbon (Fig. 2a pathway (III)) (Bailey 1982). It is also possible that some BQD was formed directly from CBZ without the involvement of ozone (Fig. 2a pathway (II). However, because BQD was formed significantly after BQM, this was not a major pathway.

TP225 was probably formed via a radical reaction from the intermediate product N,N-bis(2-formylphenyl)urea (Fig. 2b). As TP225 was formed, carbamic acid was probably formed too, but this product was not detected because carbamic acid is not stable, so it was further transformed to ammonia and carbon dioxide. TP225 could react with hydroxyl radicals to form TP241 (Fig. 2b). Because the pH of the solution during the experiments was slightly acidic, it was possible for this product to react through a Friedel-Craft mechanism to form TP223 (Fig. 2b). TP223 reacted with hydroxyl radicals to form TP239 (Fig. 2b).

**Fig. 3:**
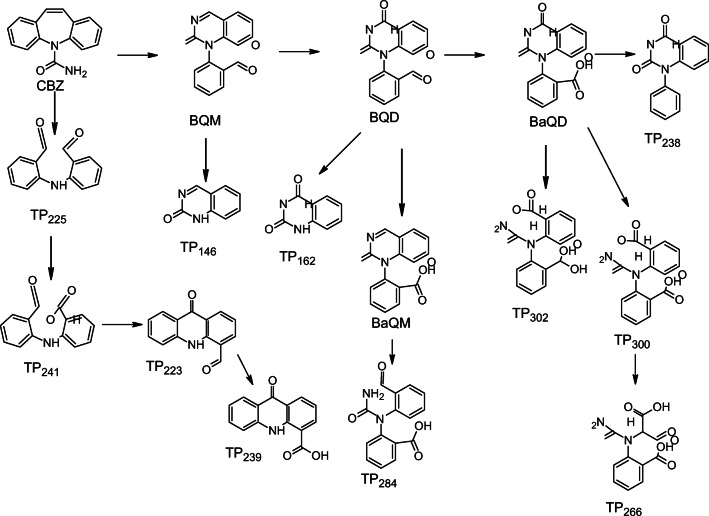
CBZ ozonation pathways

The aldehydes in both BQM and BQD could react with ozone or hydroxyl radicals to form BaQM and BaQD, respectively. TP146 and TP162 were formed via the cleavage of a nitrogen-carbon bond. The mechanism of this reaction is unknown. The ozonation of the pharmaceutical metoprolol without a radical scavenger leads to a similar bond cleavage; however, the product was not formed in the presence of a radical scavenger (Tay et al. 2013), so the reaction probably involves radicals. TP300 was formed from BaQD via a hydroxylation reaction. TP238 could be formed from BaQD via a decarboxylation reaction. The complete reaction pathway of CBZ is presented in Fig. 3.

### Quantification of CBZ ozonation products

The main products which could be detected with UV when CBZ was ozonated were BQM, BQD, and TP225 (Fig. 1a). All three products were isolated from a sample of CBZ ozonated for 30 min. Only BQD could be obtained as a pure standard.

No pure BQM standard could be obtained. The isolated sample contained both BQM and BQD. Since a pure sample of BQD was available, quantitative NMR was performed on the BQM sample. Quantitative NMR showed that the BQM sample contained 35% BQD. Subsequently, the amount of BQD in the sample was quantified with LC-UV using the BQD standard, and hence, the concentration of BQM in the standard was measured. The calibration curves for BQM and BQD were made by diluting the standards.

The stability of BQM and BQD in water was investigated by diluting the products in water and analyzing their UV peak areas. Afterwards, the sample was stored at room temperature and re-analyzed over 30 days. During that time, no decrease in the peak areas was observed, and the UV peak area for BQM and BQD varied with relative standard deviations of 9% and 14% respectively.

TP225 could also be isolated; however, the product was deemed too unstable to be used for quantification. Instead, the formation and transformation of TP225 was estimated by measuring the change in the UV peak area of TP225 as a function of time and by comparison with the results for BQM and BQD.

### Formation of CBZ ozonation products

**Fig. 4:**
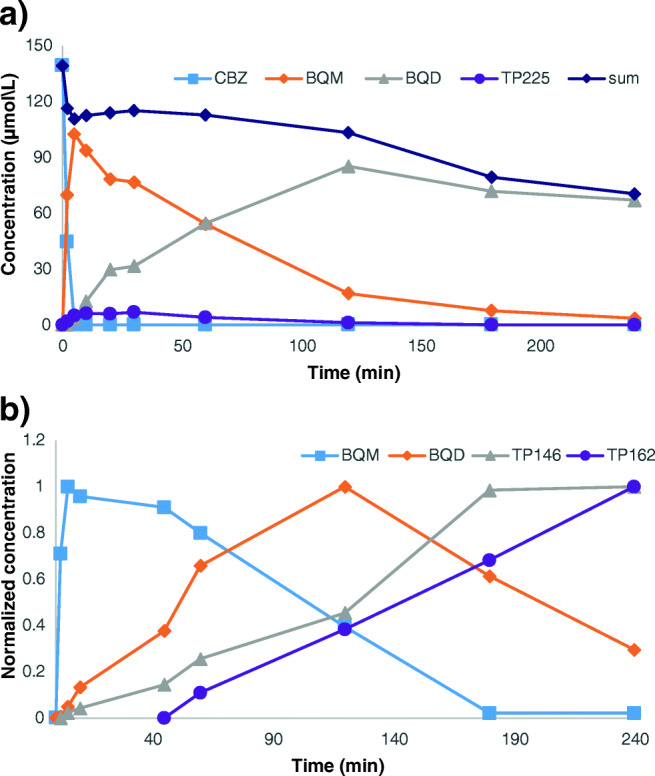
Molar concentrations (**a**) of CBZ, BQM, and BQD and normalized EIC peak areas (**b**) for BQM, BQD, TP146, and TP162

The molar concentrations of CBZ, BQM, and BQD in an uncatalyzed ozonation reaction are shown in Fig. 4a. The initial concentration of CBZ was 139 μmol/L. The maximum concentration of BQM was 103 μmol/L, so at least 74% of CBZ was transformed into BQM. BQM is much more stable than CBZ: the concentration of CBZ was below the detection limit after 5 min, while there was still 3 μmol/L BQM after 240 min of ozonation. At 120 min, the concentration of BQD was 85 μmol/L, so at least 83% of BQM was transformed into BQD. Also, BQD is more stable than CBZ. When pure BQD was ozonated, 16% of the initial concentration of BQD was still present after 240 min of ozonation.

In order to estimate the amount of TP225 which was formed, the calibration curves for CBZ and BQD were applied to TP225. Both calibration curves gave the same concentration (RSD ≤ 20%). Based on this, it was estimated that 4% of CBZ was transformed into TP225.

The sum of the concentrations of all the major products decreased with 16.5% in the first 2 min, after which the sum remained stable until 120 min. After that, the concentration of BQD started to decrease. The concentration of CBZ decreased to below the detection limit in 5 min, while both BQM and BQD were much more stable. The concentration of BQM decreased to 3.4% of the maximum concentration in 235 min.

The concentration of other products was difficult to study, since the UV and extracted ion chromatogram (EIC) peak areas for most of them were very low. However, the peak areas of TP146 and TP162 were large enough to be investigated. The breakage of the bond between nitrogen and the aromatic ring in BQM and BQD led to the formation of TP146 and TP162, respectively. These products could not be isolated and no commercial standards were available, so the formation of these products was investigated using the peak areas of the EIC. TP146 was detected after 5 min of ozonation and the concentration increased until the concentration of BQM was below the quantification limit (Fig. 4b). TP162 was detected after 60 min of ozonation and the concentration increased to the end of the experiment (Fig. 4b). Both TP146 and TP162 seemed to be minor products, because the maximum peak area for TP146 was 2% of the maximum peak area for BQM, and the maximum peak area for TP162 was 0.2% of the maximum peak area for BQD.

### Toxicity

The toxicity of CBZ and its major products were determined using the ECOSAR software. ECOSAR can be used to calculate both the acute and chronic toxicity of small compounds towards fish, daphnid, and green algae. ECOSAR has shown excellent correlation between calculated and measured toxicity (Öberg, 2004). The acute toxicity of most of the ozonated products was lower than CBZ. The exceptions were BQD and TP162. The chronic toxicity of BQM, BQD, TP225, and TP162 were more toxic than CBZ. A more detailed description of the method and the results can be found in the supplementary information.

## Conclusions

CBZ reacted quickly with ozone, and could no longer be detected after 5 min. CBZ underwent a direct ozonation reaction to BQM. Approximately, 74% of CBZ was transformed into BQM. BQM also reacted through direct ozonation to form BQD. Approximately, 83% of BQM was transformed into BQD. BQM reacted much more slowly with ozone than CBZ. Additionally to BQM and BQD, 13 different products were detected. None of the products detected in this study kept the tricyclic structure of CBZ. After BQM and BQD, the product with the highest concentration was TP225. TP225 was isolated and the structure was confirmed with NMR. It was estimated that 4% of CBZ transformed into TP225. BQM reactesd further to TP146 via the breakage of the bond between nitrogen and the aromatic ring and to BaQM via the oxidation of the aldehyde group to a carboxylic acid. Similarly, BQD formed TP162 and BaQD. BaQM and BaQD could further be transformed via ring opening reactions. One of the aldehydes in TP225 was oxidized to a carboxylic acid which then underwent a ring closing reaction. After this, the remaining aldehyde was oxidized to a carboxylic acid.

## Electronic supplementary material

ESM 1 (DOCX 1600 kb)
