# Supplementary material for: Ozonation of carbamazepine and its main transformation products: product determination and reaction mechanisms
Source: Environ Sci Pollut Res Int. 2020 Apr 25;27(18):23258–69. doi: 10.1007/s11356-020-08795-0 (PMC7293669; doi:10.1007/s11356-020-08795-0)
Supplement: Supplementary file 1 — (DOCX 1600 kb) [file 11356_2020_8795_MOESM1_ESM.docx]

SUPPLEMENTARY INFORMATION

**Ozonation of carbamazepine: product determination and reaction mechanisms**

**Matilda Kråkström^a^, Soudabeh Saeid^b^, Pasi Tolvanen^b^, Narendra Kumar^b^, Tapio Salmi^b^ Leif Kronberg^a^, Patrik Eklund^a^**

*^a^ Laboratory of Organic Chemistry, Johan Gadolin Process Chemistry Centre, Åbo Akademi University, Biskopsgatan 8, FI-20500 Åbo/Turku, Finland*

*^b^ Laboratory of Industrial Chemistry and Reaction Engineering, Johan Gadolin Process Chemistry Centre, Åbo Akademi University, Biskopsgatan 8, FI-20500 Åbo/Turku, Finland*

**Table S1. Mass spectrometer parameters**

| Parameter | Value (ion trap) | Value (QToF) |
| --- | --- | --- |
| End plate offset | - | -500 V |
| Capillary | 140.2 V | 4500 V |
| Nebulizer | 40 psi | 1,6 bar |
| Skimmer | 40 V | - |
| Drying gas | 8 L/min | 8 L/min |
| Drying temperature | 350 °C | 200 °C |
| Trap drive | 27.0 | - |
| Transfer ion funnel 1 and 2 RF | - | 200 Vpp |
| Octapole/hexapole RF | 132 Vpp | 80 Vpp |
| Quadrupole ion energy | - | 3 eV |
| Quadrupole low mass | - | 100 m/z |
| Collision energy | - | 7 eV |
| Collision RF | - | 150 Vpp |
| Transfer time | - | 109 µs |
| Prepulse storage | - | 1. µs |

**Method validation**

The limit of quantification (LOQ) was estimated using visual inspection of calibration samples with concentrations close to the LOQ. The linearity was calculated for concentrations between LOQ and 50 mg/L (the maximum concentration of CBZ was 30 mg/L). The intra-day precision was calculated by injecting the same sample three times during the same day. The inter-day precision was calculated by preparing samples using the method presented in section 2.2. five times on different days and comparing the resulting peak areas. The results for precision are presented as the relative standard deviation of the peak areas. The accuracy was calculated by comparing the expected results for five samples prepared according to section 2.2. (i.e. 30 mg/L for CBZ and 6 mg/L for BQD) with the calculated results. The average difference between the expected result and the calculated result was divided by the expected result to obtain the relative standard deviation. The LOQ was 70 µg/L of CBZ, 100 µg/L of BQM and 90 µg/L for BQD. The linearity was 0.999 for CBZ, 0.998 for BQM and 0.999 for BQD. The intra-day precision was 5 % for CBZ, 8 % for BQM and 6 % for BQD. The inter-day precision was 9 % for CBZ, 11 % for BQM and 9 % for BQD. The accuracy was 6 % for CBZ and 4 % for BQD.

**Mass spectra of identified products**


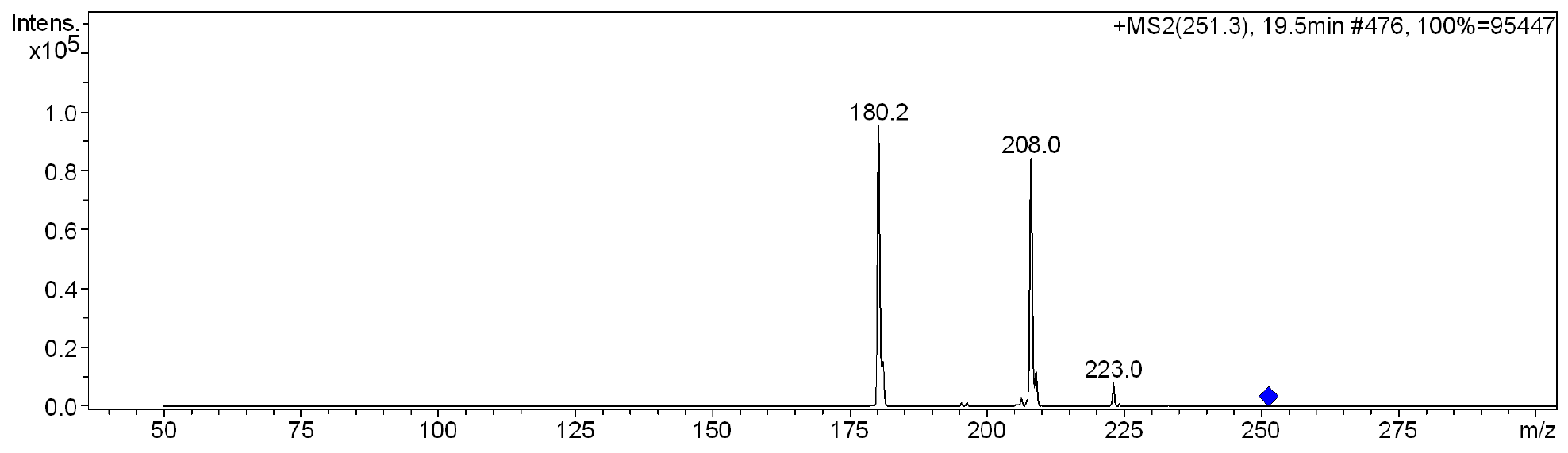


Figure S1. MS^2^ spectra of BQM


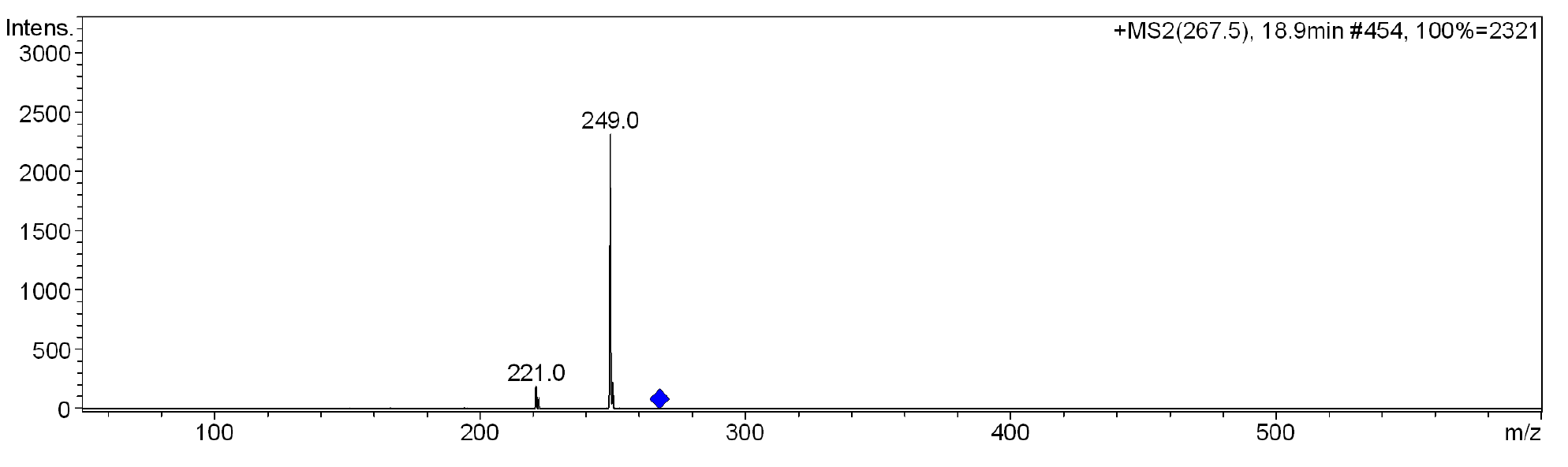


Figure S2. MS^2^ spectra of BaQM


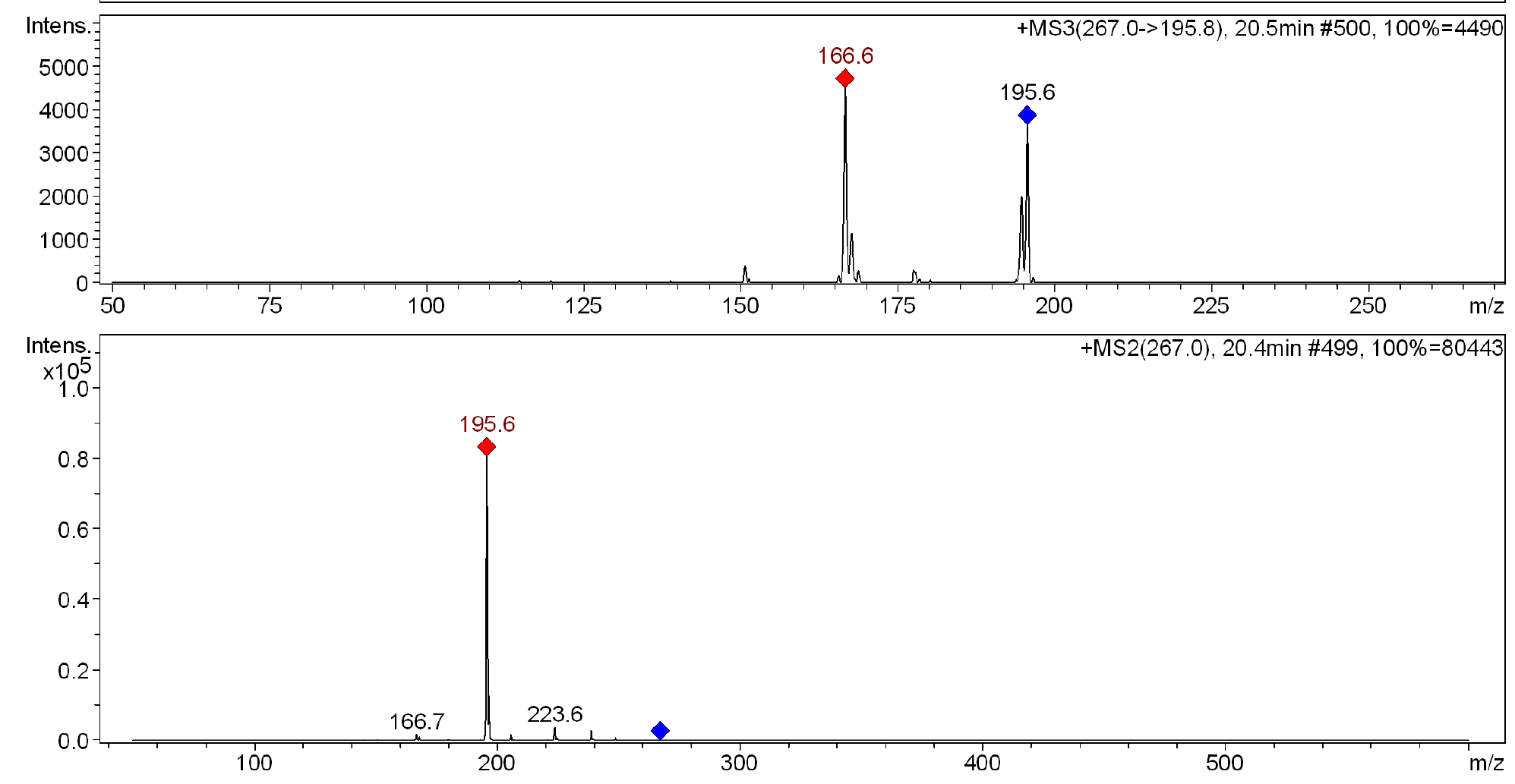


Figure S3. MS^2^ (above) and MS^3^ (below) spectra of BQD


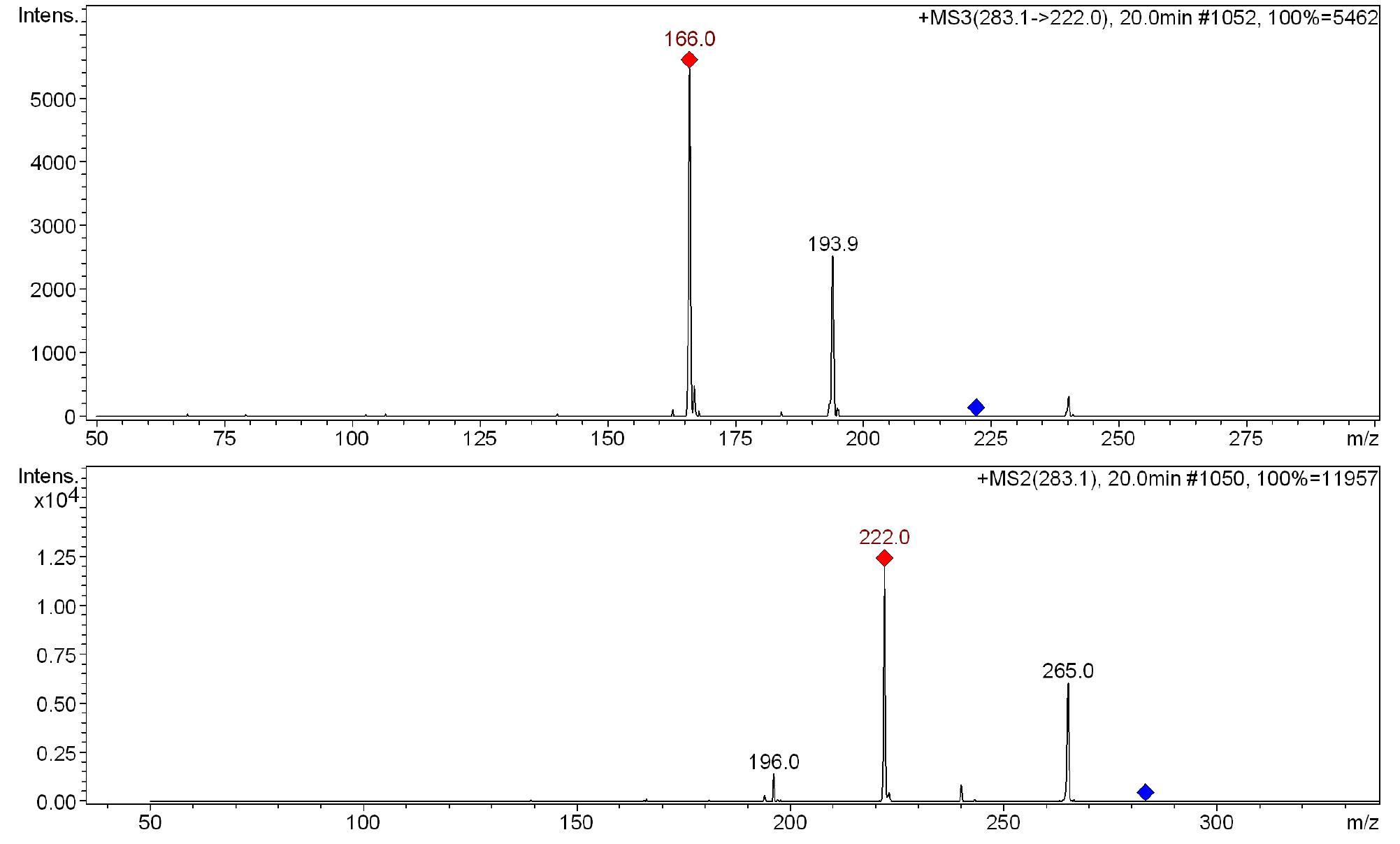


Figure S4. MS^2^ (above) and MS^3^ (below) spectra of BaQD


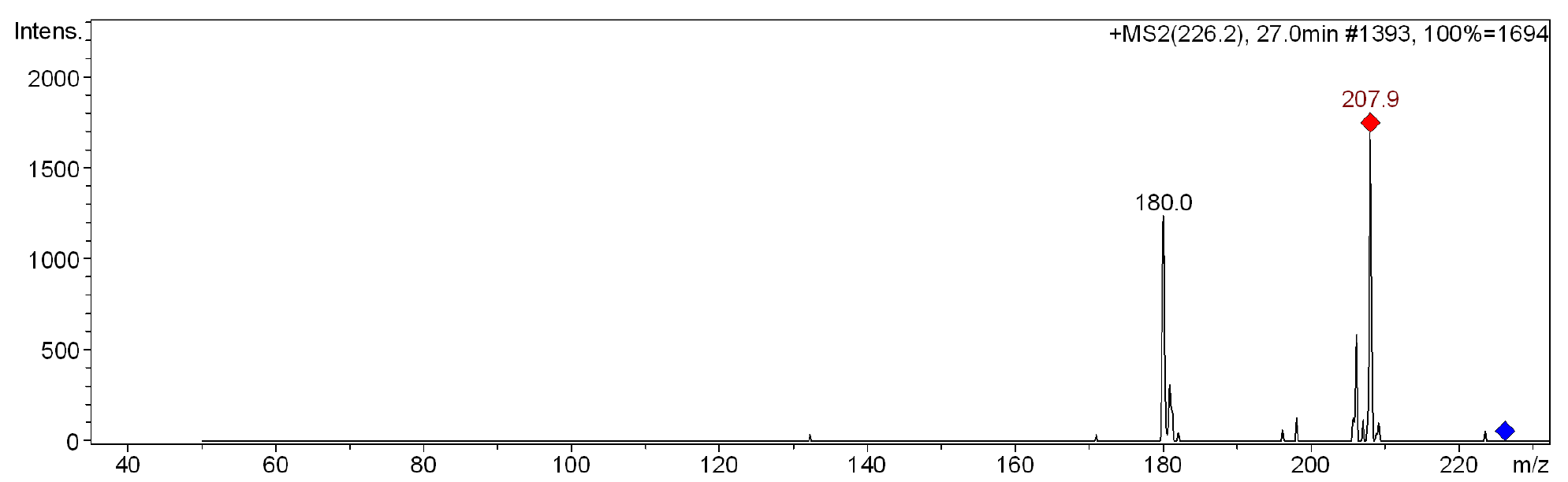


Figure S5. MS^2^ spectra of TP225


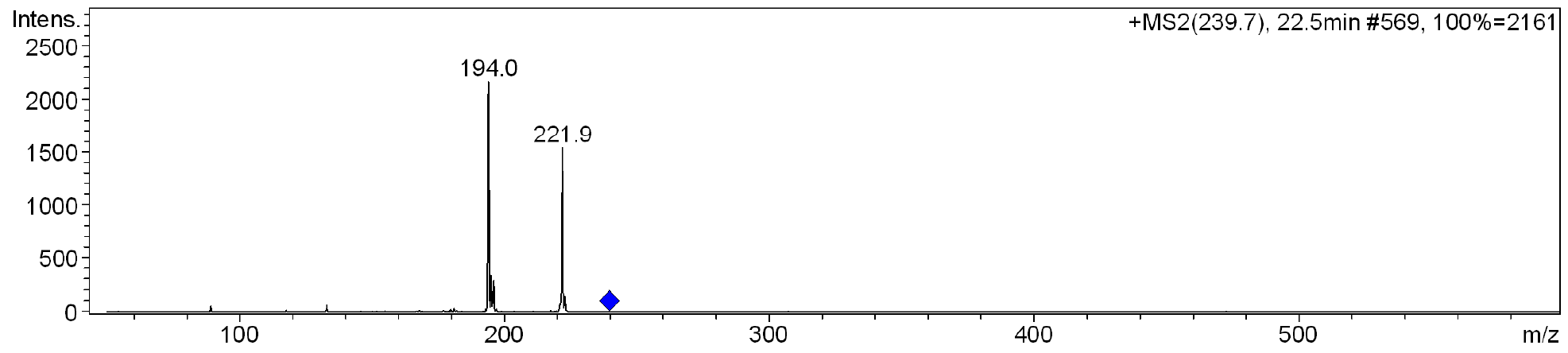


Figure S6. MS^2^ spectra of TP239


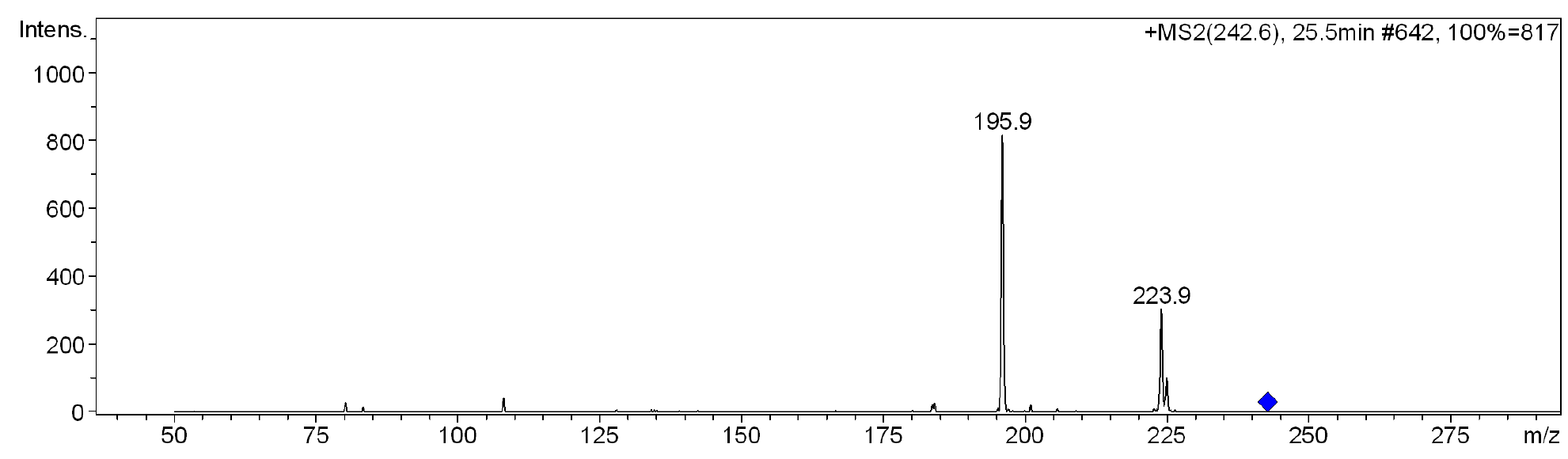


Figure S7. MS^2^ spectra of TP241


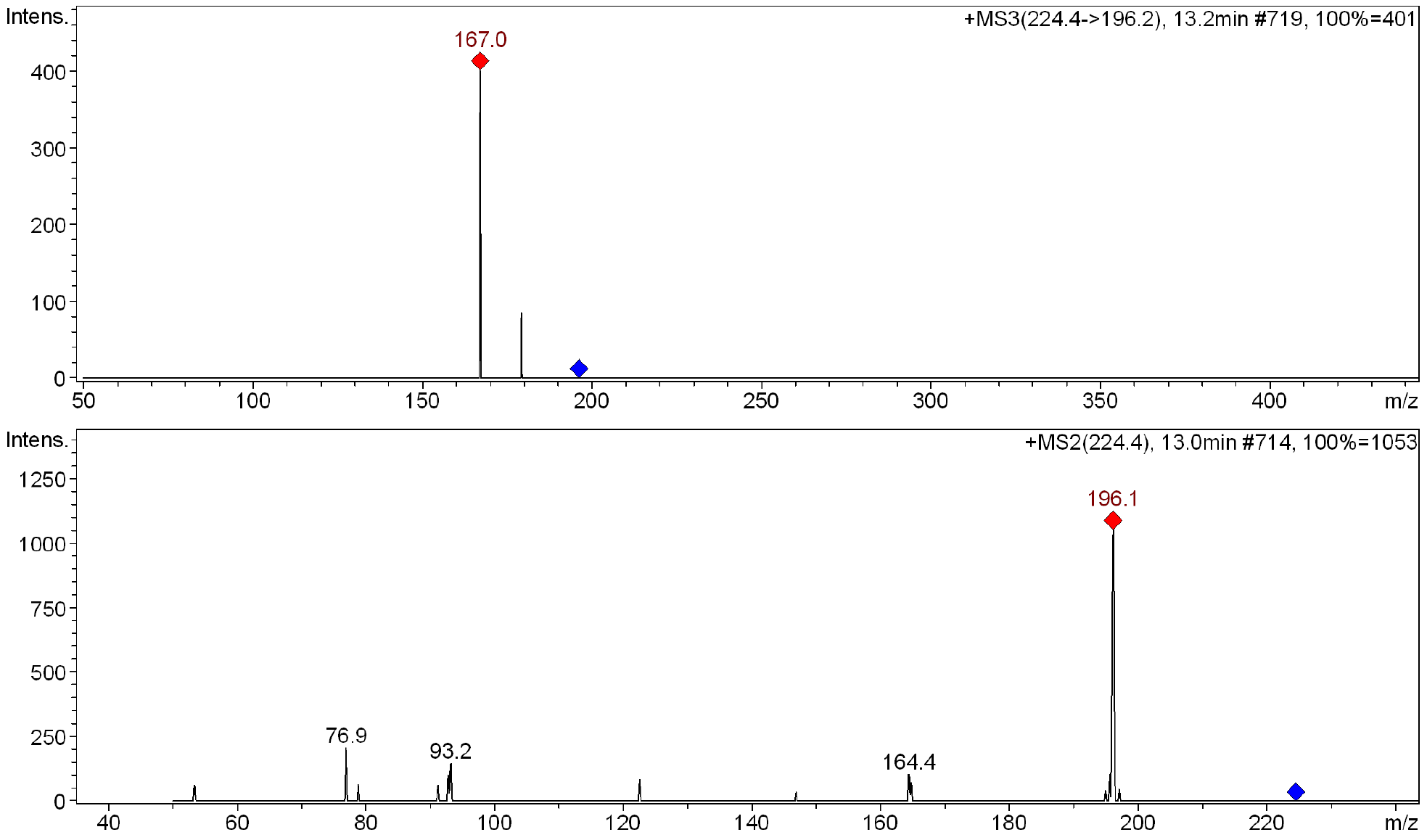


Figure S8. MS^2^ (above) and MS^3^ (below) spectra of TP223


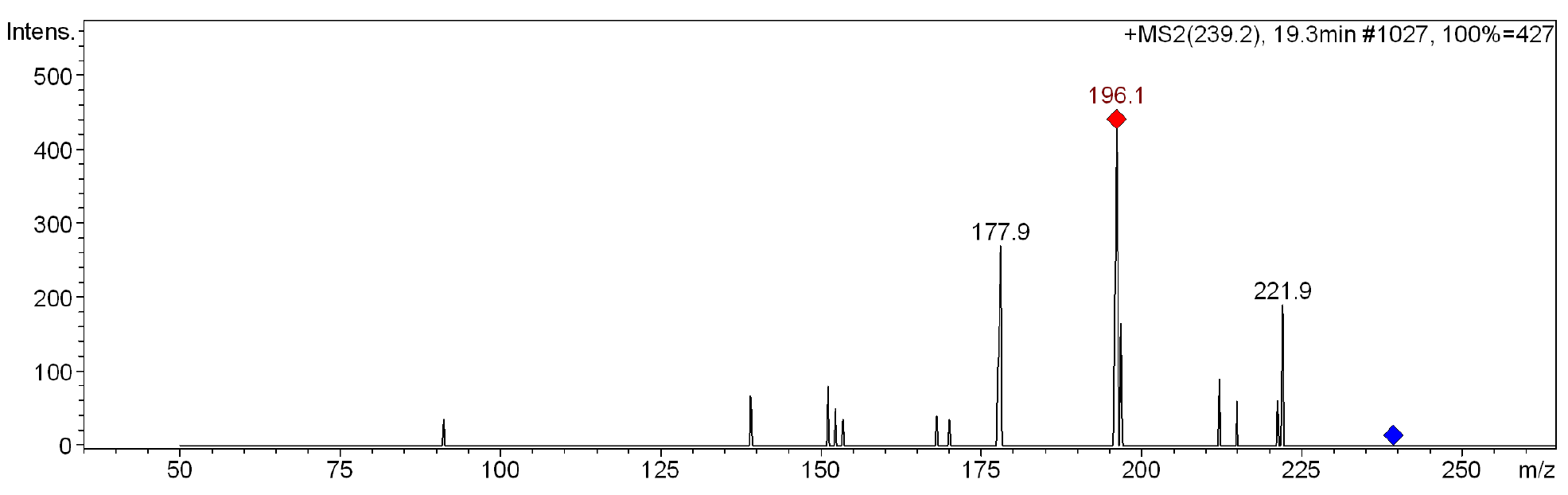
Figure S9. MS^2^ spectra of TP238


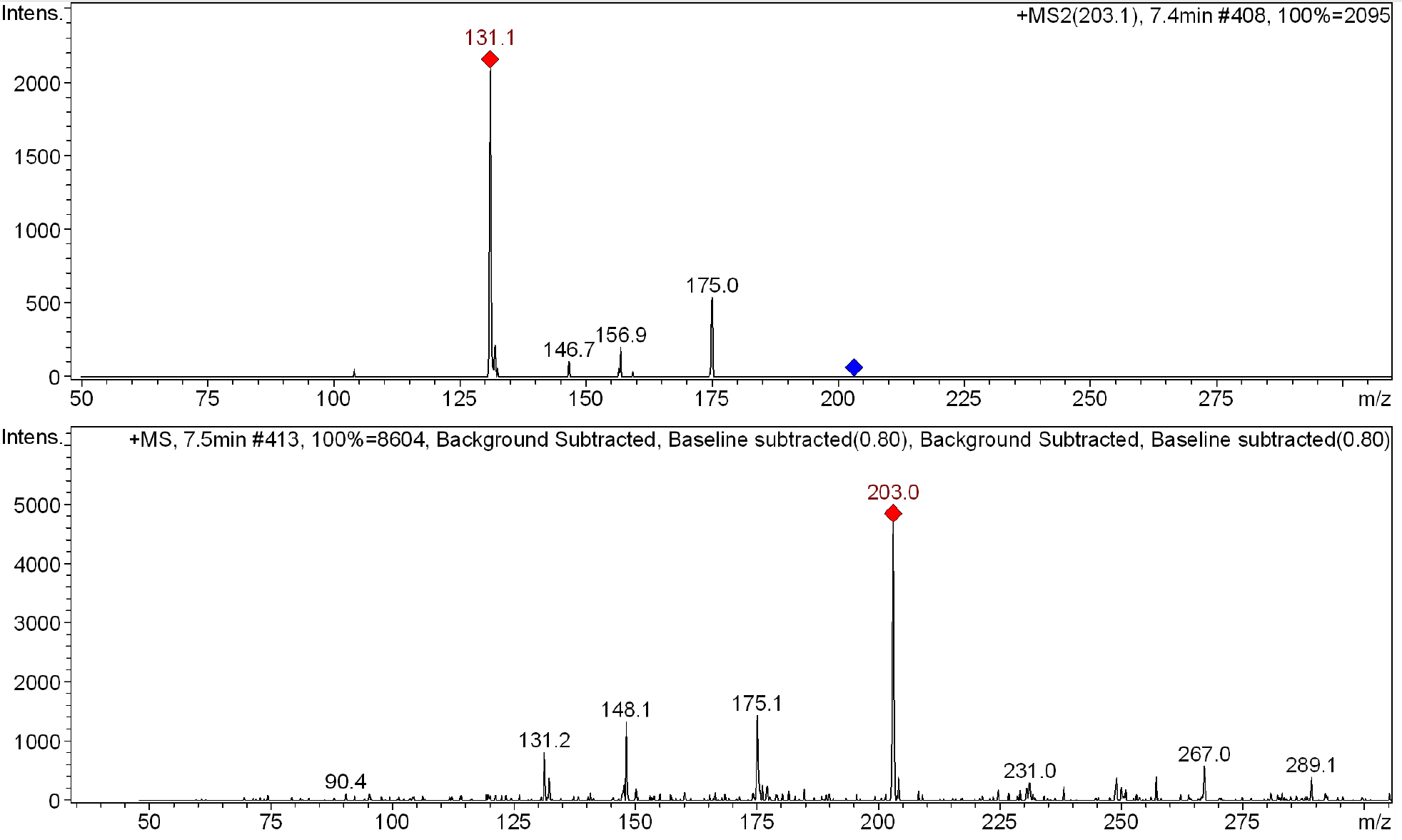


Figure S10. MS^2^ (above) and MS^3^ (below) spectra of TP266


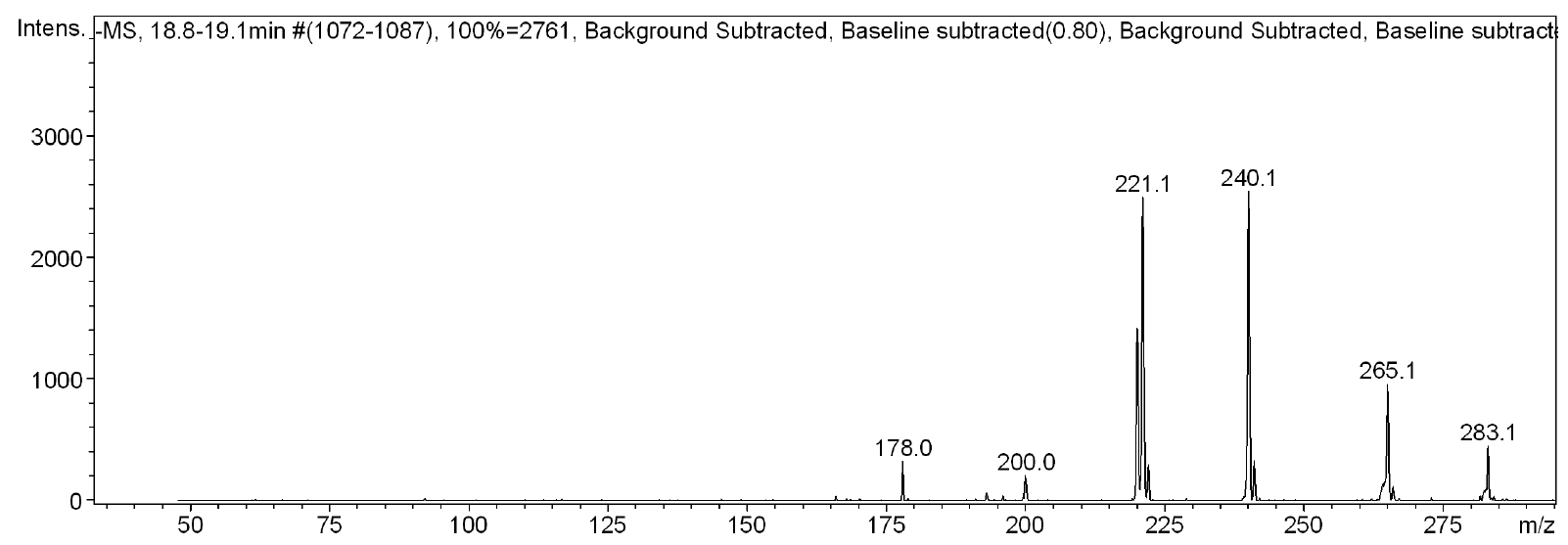


Figure S11. Full scan spectra of TP284


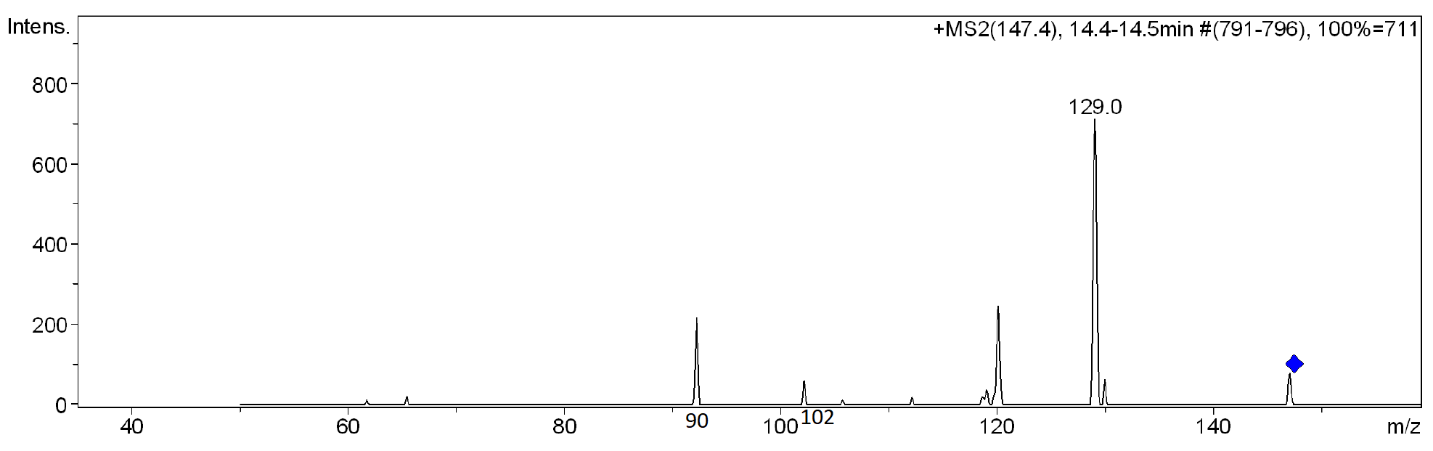


Figure S12. MS^2^ spectra of TP146


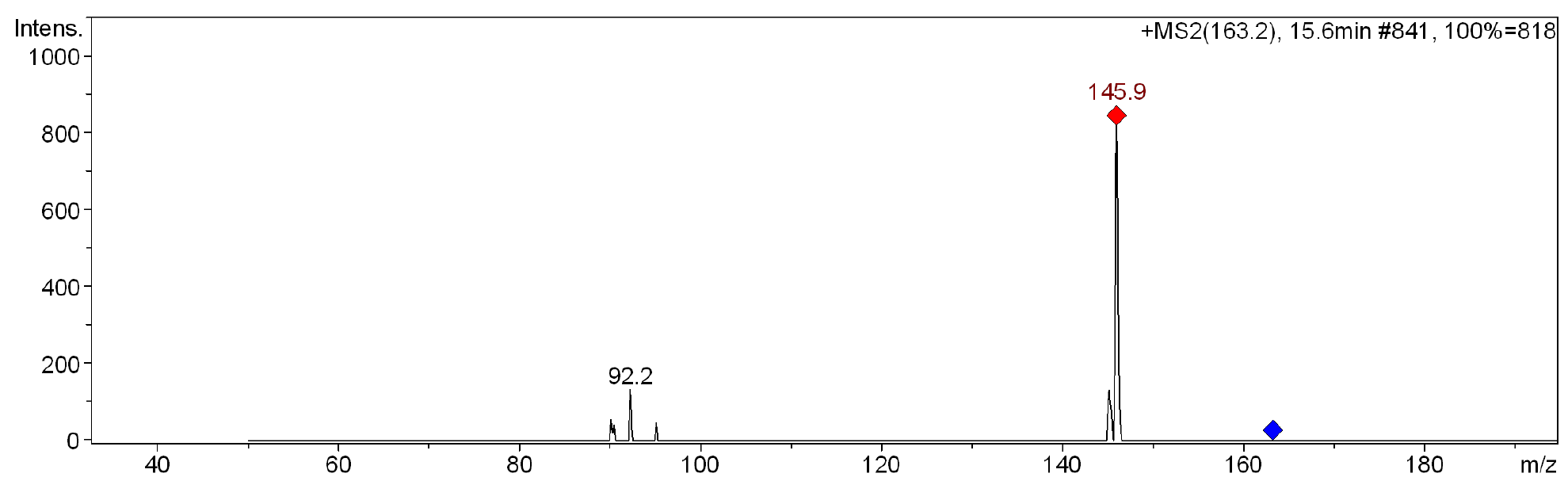


Figure S13. MS^2^ spectra of TP162


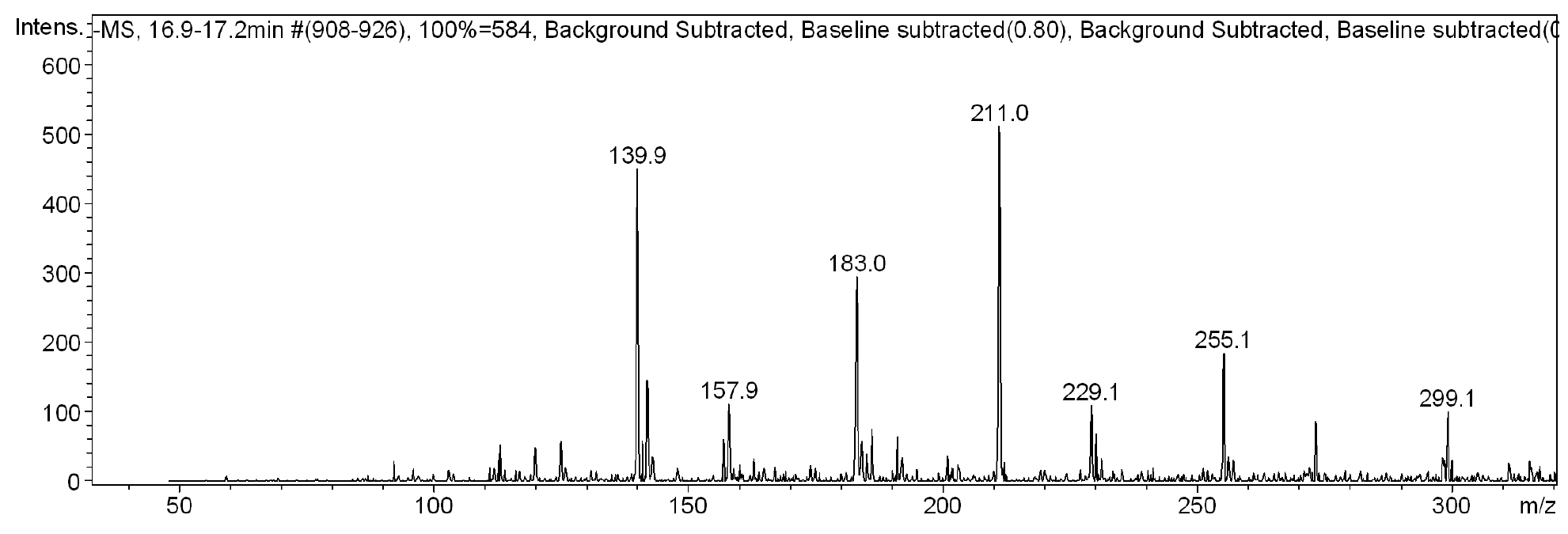


Figure S14. MS scan of TP300


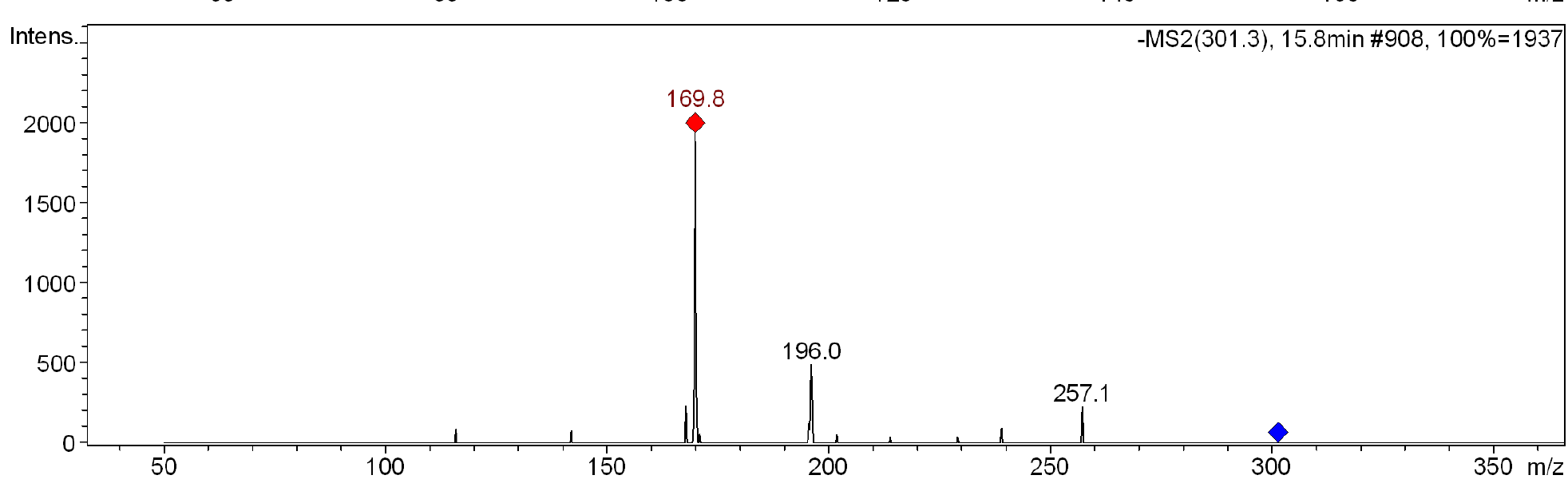


Figure S15. TP302


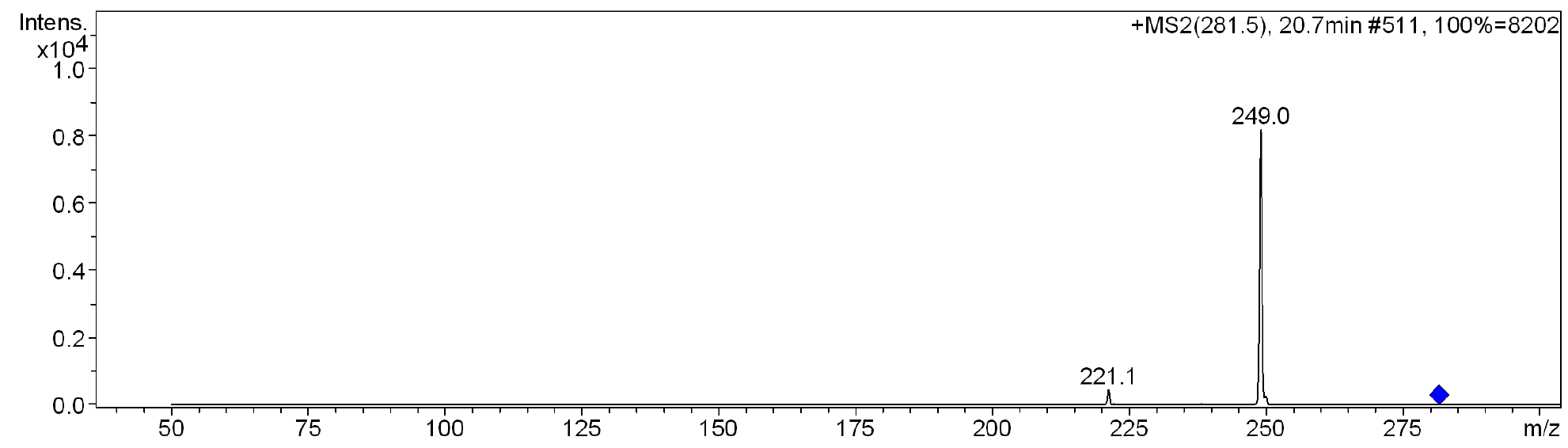


Figure S16. MS^2^ spectra of MeOBQM


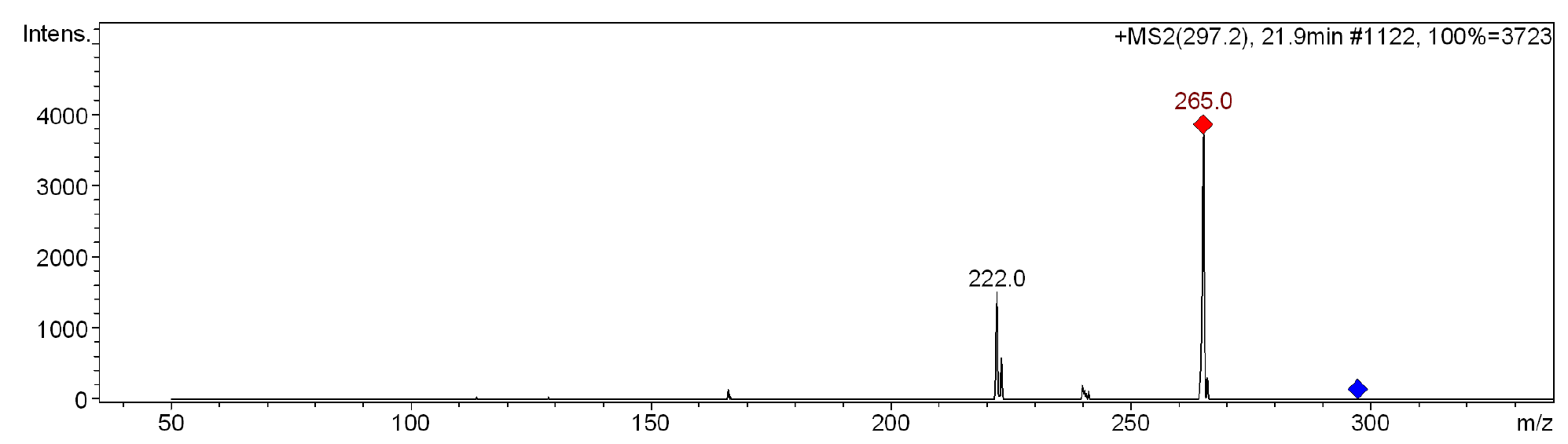


Figure S17. MS^2^ spectra of MeOBQD

**Identification of previously published products**

Two of the main CBZ ozonation products (BQM and BQD) have previously been identified with MS and NMR (Hu et al. 2009). BQM has [M+H]^+^ = 251 and two main fragments with m/z = 180 and 208. BQD has [M+H]^+^ = 267 and two main fragments with m/z = 196 and 167 (McDowell et al. 2005). Both products were identified also in this study. The structures of BQM and BQD were confirmed using isolated standards. The NMR spectra matched the spectral data published by McDowell et al. (2005). BQM is also formed during dielectric barrier discharge oxidation (Liu et al. 2016) and solar photolysis (Yang et al. 2016). BQD is also formed during phototransformation (Kosjek et al. 2009, de Laurentiis et al. 2012, Yang et al. 2016, Rao et al. 2013), biotransformation (Golan-Rozen et al. 2015), chloramination (Han et al. 2018) and dielectric barrier discharge oxidation (Liu et al. 2012).

Other previously detected CBZ ozonation products include the hydroxylated derivatives formed from BQM and BQD: BaQM ([M+H]^+^ = 267 and main fragments with m/z = 249 and 221) and BaQD ([M+H]^+^ = 283 and main fragments with m/z = 265 and 222). BaQM is formed during catalytic oxidation (Hu et al. 2009), ozonation (Azaïs et al. 2017, Hübner et al. 2014) and phototransformation (rao et al. 2013). BaQD is formed during ozonation (Azaïs et al. 2017, McDowell et al. 2005, Dwivedi et al. 2018, Hübner et al. 2014, Liu et al. 2012), biotransformation (Golan-Rozen et al. 2015) and phototransformation (Li et al. 2011).

TP239 ([M+H]^+^ = 240 and main fragment with m/z = 222) (Pan et al. 2017) is formed during catalytic ozonation (24). In this study, the MS^2^ spectrum also showed a fragment with m/z = 194, corresponding to the loss of a COOH group. TP223 ([M+H]^+^ = 224 and main fragments with m/z = 196 and 167) (Hübner et al. 2014) is formed during phototransformation (Yang et al. 2016, Rao et al. 20213, Duan et al. 2019), catalytic oxidation (Hu et al. 2009), ozonation (Hübner et al. 2014) and biotransformation (Li et al. 2013). TP146 ([M+H]^+^ = 147 and main fragments with m/z = 129, 120 and 92) and TP162 ([M+H]^+^ = 163 and main fragments with m/z = 146 and 90) have been identified as CBZ ozonation products (Azaïs et al. 2017). These products are formed from the breakage of the C-N bond in BQM and BQD, respectively. In this study, the MS^2^ spectrum of TP162 also showed a fragment with m/z = 118, corresponding to the loss of CH_3_NO. Due to the presence of methanol in the experiments, it was possible to detect methoxylated versions of BQM ([M+H]^+^ = 281) and BQD ([M+H]^+^ = 297). These products are also formed during biotransformation (Golan-Rozen et al. 2015).

**^1^H NMR spectra of BQM and BQD**


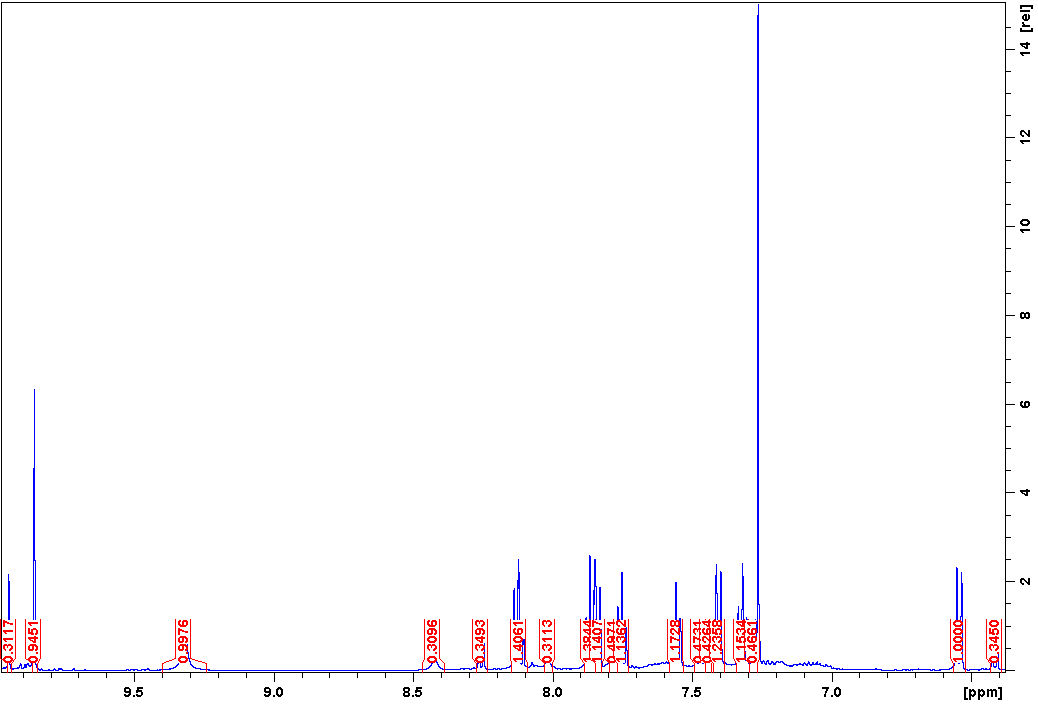


Figure S18. ^1^HNMR spectrum of BQM


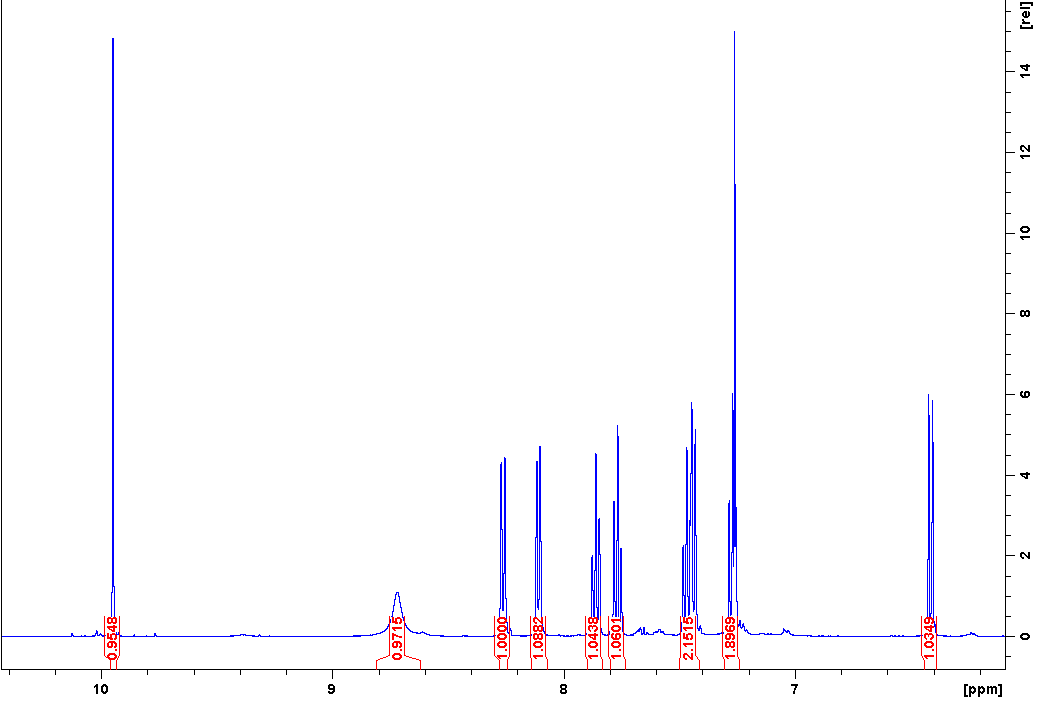


Figure S19. ^1^HNMR spectrum of BQD

**Identification of TP225**

The 1H NMR spectrum of TP225 can be seen in figures S19 and S20. In addition to TP225, a methylated product of TP225 was present during the NMR analysis. The methylated structure shows similar shifts to those of TP225. However, it has a singlet at 5.4 (carbon shift 100.9) instead of 10.05 (carbon shift 192.9) and an additional singlet at 3.4 (carbon shift 53.4), indicating the presence of a hemiacetal instead of an aldehyde (figure 2). This structure was probably formed during the sample work up when TP225 was dissolved in MeOH. When the purified sample was dissolved in water and analyzed with MS using water with 0.1 % formic acid as an eluent, a product with the accurate mass 264.0607 was detected in addition to TP225, which corresponds to the empirical formula C_14_H_11_NNaO_3_ with an error of 11 ppm. This indicates that when the product is dissolved in water the MeOH group is lost and the aldehyde group is transformed to a carboxylic acid.

The fragmentation pathway of TP225 was investigated by using the ion trap mass spectrometer operating in MS^n^ mode. TP 225 has the fragmentation pathway 226 -> 208 -> 206 -> 178. The pathway likely involves a McLafferty rearrangemen (Figure 3). The fragment with m/z 178 further fragments to m/z = 151 and 77.


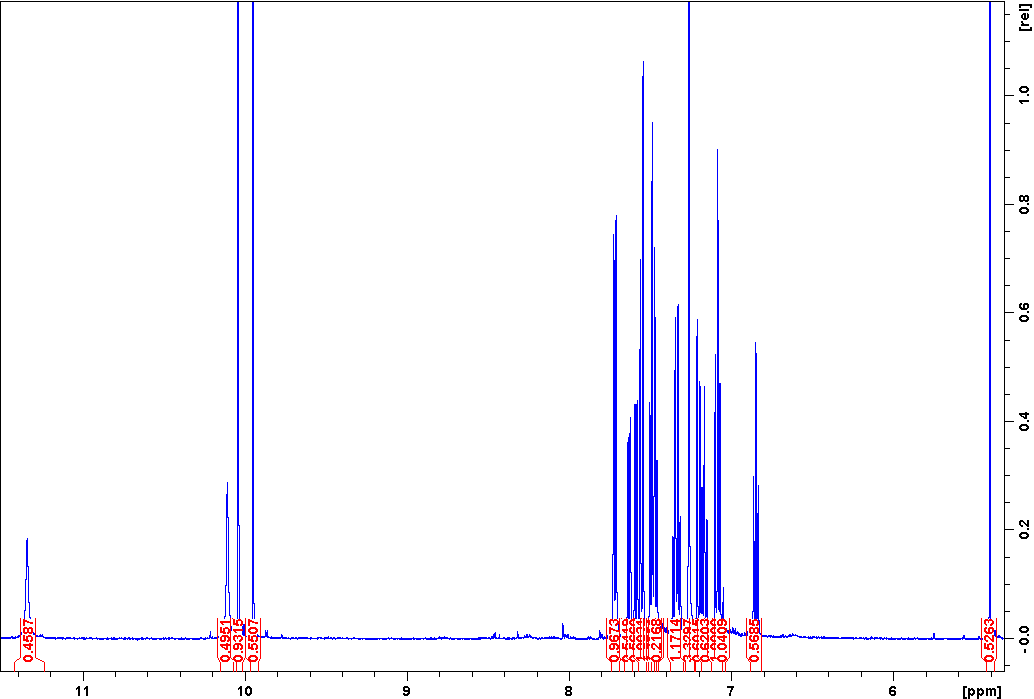


Figure S20. ^1^HNMR spectrum of TP225


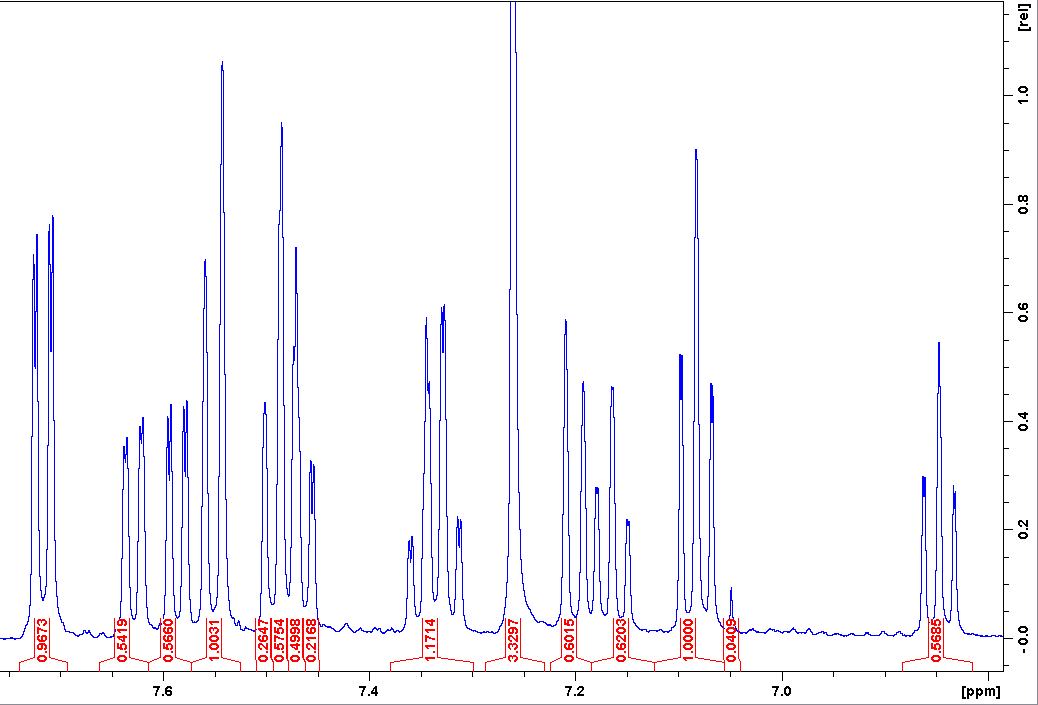


Figure S21. Aromatic region of the ^1^HNMR spectrum of TP225





Figure S22. suggested ^1^H NMR and ^13^C NMR shifts for TP225 a) and b) and

MeOTP225 c) and d)

Figure S23. The fragmentation pathway of TP225


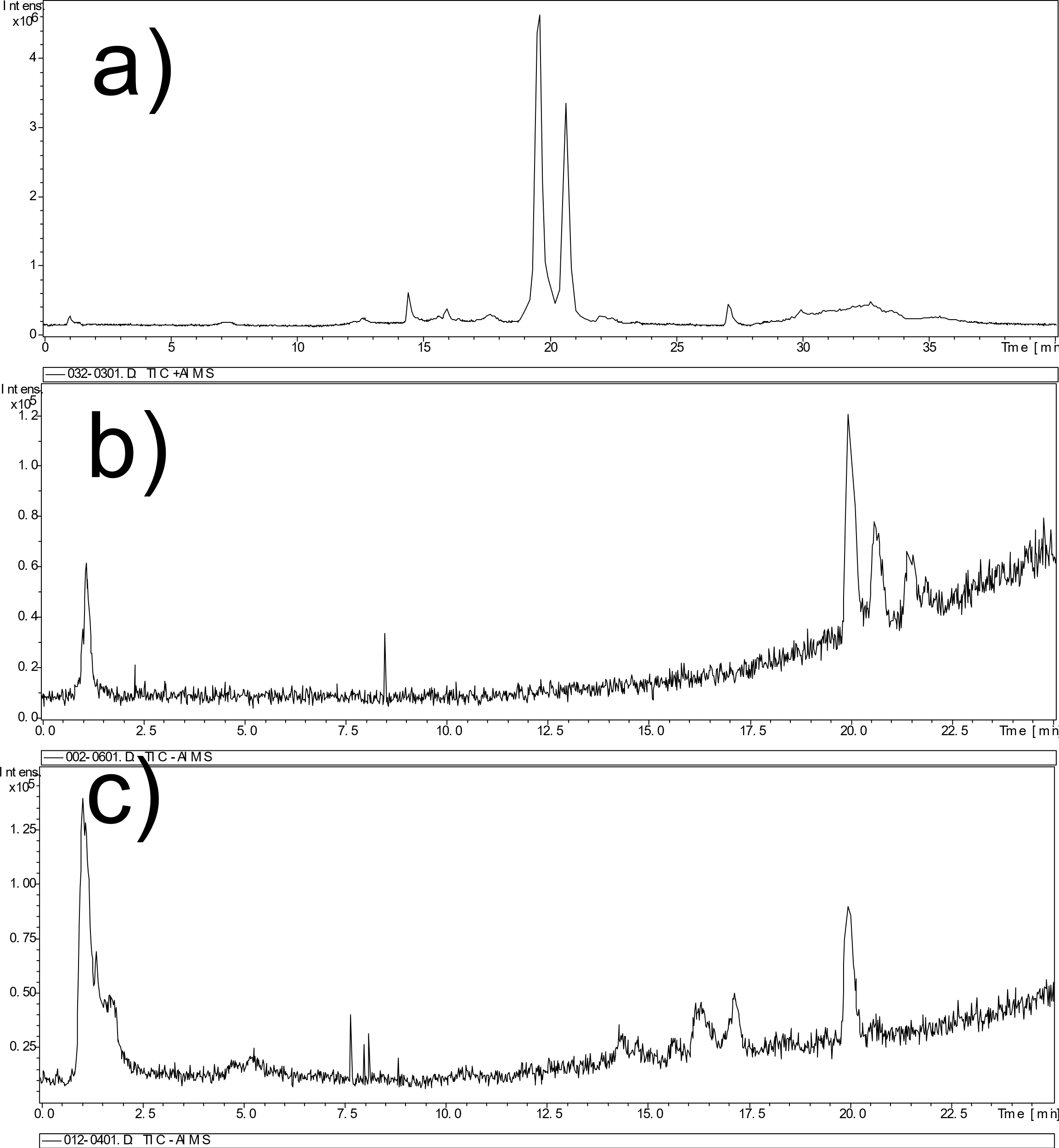


Figure S24. TIC of a) a CBZ ozonation sample taken after 10 min b) a BQD sample taken after 0 min and c) a BQD sample taken after 240 min of ozonation.

**Toxicity**

The toxicity of CBZ and its major products was determined using the ECOSAR software. ECOSAR can be used to calculate both the acute and chronic toxicity of small compounds towards fish, daphnid and green algae. ECOSAR has shown excellent correlation between calculated and measured toxicity (Öberg 2004). The acute toxicity of CBZ and its ozonation products was highest towards green algae (Table 1). The toxicity of most of the ozonated products was lower than CBZ. The exceptions were BQD and TP162. The chronic toxicity calculations showed that different species were most sensitive for the different products (Table 1). BQD, TP225 and TP162 were more toxic than CBZ towards fish, BQM, BQD and TP225 were more toxic than CBZ towards daphnid and BQD and TP162 were more toxic towards green algae. This is a concern since the CBZ ozonation products are more stable towards ozonation than CBZ.

Table 1. Acute and chronic toxicity of CBZ and its ozonation products calculated using the ECOSAR software.

|  | | Acute toxicity (mg/L) | | | Chronic toxicity (mg/L) | | |
| --- | --- | --- | --- | --- | --- | --- | --- |
| Compound | Class | Fish | Daphnid | Algae | Fish | Daphnid | Algae |
| CBZ | Substituted ureas | 40.9 | 14.1 | 0.26 | 1.05 | 1.17 | 0.096 |
| BQM | aldehydes (mono) | 29.3 | 28.3 | 15.6 | 5.59 | 0.284 | 5.59 |
| BQD | aldehydes (mono) | 35.9 | 35.1 | 19 | 7.29 | 0.343 | 6.69 |
|  | carbonyl ureas | 99.4 | 22.1 | 0.021 | 0.59 | 5.03 | 0.0055 |
| BaQM | neutral organics | 659 | 395 | 367 | 68.6 | 44.7 | 108 |
| BaQD | carbonyl ureas | 215 | 37.6 | 0.243 | 1.38 | 7.15 | 0.059 |
| TP225 | aldehydes (poly) | 2.38 | 2.37 | 0.547 | 0.078 | 0.201 | 0.236 |
| TP146 | neutral organics | 1140 | 584 | 287 | 98.7 | 43.1 | 60.2 |
| TP162 | carbonyl ureas | 118 | 29 | 0.013 | 0.677 | 7.1 | 0.0033 |
| TP300 | substituted ureas | 75.5 | 17.1 | 1.35 | 2.27 | 2.48 | 0.514 |
